# Insulin resistance and glycemic abnormalities are associated with deterioration of left ventricular diastolic function: a cross-sectional study

**DOI:** 10.1186/1475-2840-9-63

**Published:** 2010-10-15

**Authors:** Wilfried Dinh, Mark Lankisch, Werner Nickl, Daniel Scheyer, Thomas Scheffold, Frank Kramer, Thomas Krahn, Rolf M Klein, Michael Coll Barroso, Reiner Füth

**Affiliations:** 1 Institute for Heart and Circulation Research, University Witten/Herdecke, Germany; 2 Helios Clinics Wuppertal, Department of Cardiology, Wuppertal, Germany; 3 CoroVital, Institute for Sports Medicine, Wuppertal, Germany; 4 Stiftung Institut für Herzinfarktforschung Ludwigshafen (IHF), Germany; 5 Global Biomarker Research, Bayer Schering Pharma, Wuppertal, Germany; 6 Augusta Hospital Düsseldorf, Department of Cardiology, Düsseldorf, Germany

## Abstract

**Background:**

Left ventricular diastolic dysfunction (LVDD) is considered a precursor of diabetic cardiomyopathy, while insulin resistance (IR) is a precursor of type 2 diabetes mellitus (T2DM) and independently predicts heart failure (HF). We assessed whether IR and abnormalities of the glucose metabolism are related to LVDD.

**Methods:**

We included 208 patients with normal ejection fraction, 57 (27%) of whom had T2DM before inclusion. In subjects without T2DM, an oral glucose tolerance test (oGTT) was performed. IR was assessed using the Homeostasis Model Assessment of Insulin Resistance (HOMA-IR). The lower limit of the top quartile of the HOMA-IR distribution (3.217) was chosen as threshold for IR. LVDD was verified according to current guidelines.

**Results:**

IR was diagnosed in 38 (18%) patients without a history of diabetes. The prevalence of LVDD was 92% in subjects with IR vs. 72% in patients without IR (n = 113), respectively (p = 0.013). In the IR group, the early diastolic mitral inflow velocity (E) in relation to the early diastolic tissue Doppler velocity (averaged from the septal and lateral mitral annulus, E'av) ratio (E/E'av) was significantly higher compared to those without IR (9.8 [8.3-11.5] vs. 8.1 [6.6-11.0], p = 0.011). This finding remains significant when patients with IR and concomitant T2DM based on oGTT results were excluded (E/E'av ratio 9.8 [8.2-11.1)] in IR vs. 7.9 [6.5-10.5] in those without both IR and T2DM, p = 0.014). There were significant differences among patients with and without LVDD regarding the HOMA-IR (1.71 [1.04-3.88] vs. 1.09 [0.43-2.2], p = 0.003). The HOMA-IR was independently associated with LVDD on multivariate logistic regression analysis, a 1-unit increase in HOMA-IR value was associated with an odds ratio for prevalent LVDD of 2.1 (95% CI 1.3-3.1, p = 0.001). Furthermore, the E/E'av ratio increases along the glucose metabolism status from normal glucose metabolism (7.6 [6.2-10.1]) to impaired glucose tolerance (8.8 [7.4-11.0]) and T2DM (10.5 [8.1-13.2]), respectively (p < 0.001).

**Conclusions:**

Insulin resistance is independently associated with LVDD in subjects without overt T2DM. Patients with IR and glucose metabolism disorders might represent a target population to prevent the development of HF. Screening programs for glucose metabolism disturbances should address the assessment of diastolic function and probably IR.

## Background

Heart failure (HF) is increasingly common worldwide with an estimated prevalence of 2-3% [1]. It has been recognized that a large percentage of patients presenting with HF have a normal left ventricular ejection fraction (diastolic heart failure or "heart failure with normal ejection fraction", HFnEF), a condition remaining frequently undiagnosed in clinical practice. Recent data suggest that morbidity and mortality from HFnEF is nearly equal to that of systolic HF [2,3]. In the general population, which was mostly free of clinical signs of HF, left ventricular diastolic dysfunction (LVDD), the precursor of diastolic HF, was a powerful and independent predictor of death [4].

Comparable to chronic HF, type 2 diabetes mellitus (T2DM) has reached epidemic proportions, with an estimated further increase in worldwide prevalence [5]. Studies have identified diabetes as a powerful and independent risk factor for the development and prognosis of HF [6], referred to as diabetic cardiomyopathy [7]. Several studies have demonstrated left ventricular diastolic dysfunction (LVDD) to represent the first manifestation of myocardial involvement in diabetes [8-10], which is to be a key component of diabetic cardiomyopathy. Furthermore, LVDD can precede the development of diabetes [11], suggesting that LVDD is not exclusively a complication of diabetes but rather a coexisting condition.

The development of diabetic cardiomyopathy is likely multifactorial, with putative mechanisms including metabolic disturbance, changes in the extracellular matrix (ECM) components, small vessel disease, autonomic dysfunction and insulin resistance (IR). Insulin resistance may precede diabetes by a decade or more and is a pathogenic factor for T2DM [12]. Furthermore, IR has been shown to be an independent predictor of cardiovascular disease in T2DM [13] and predicted systolic HF incidence independently of established risk factors including diabetes in the community [14].

Little is known about the interactions of IR and LVDD, both frequently overlooked but nevertheless serious comorbidities of subjects with known or suspected heart disease. The aim of the present study was to explore the possible link between LVDD, IR and glucose metabolism disturbances in patients with suspected or known heart disease using definitions considering the current guidelines for the diagnosis of LVDD and glucose metabolism disorders.

## Methods

### Study population

Two hundred-eight consecutive hospitalized subjects referred to elective coronary angiography for stable or suspected coronary artery disease (CAD) were enrolled in this ongoing study. Patients with the need for coronary revascularisation either with angioplasty or coronary bypass surgery were excluded from further analysis. The protocol was approved by the local Ethics Committee, and signed informed consent was obtained from all patients. Inclusion criteria were scheduled coronary angiography and age 18-80 years. Exclusion criteria were known CAD with progressive chest pain within the last month, coronary angioplasty < 6 weeks, hypertrophic cardiomyopathy, moderate-to-severe valvular heart disease, uncontrolled hypertension, atrial fibrillation or other severe arrhythmias, or serum-creatinine > 2.5mg/dl. In patients without diabetes, a standardized oral glucose tolerance test (oGTT) was performed (75g glucose) according to the World Health Organization protocol as previously described [15]. Body mass index (BMI) was calculated as weight (kg)/height (m^2^). Abdominal girth was measured around the abdomen at the level of the belly button, and hip girth was measured at the level of maximal protrusion of the gluteal muscles.

### Echocardiography

Echocardiography for the diagnosis of LVDD was performed using a standard ultrasound system (Vingmed Vivid 7, General Electric, Milwaukee, Wisconsin). Left ventricular ejection fraction (EF) was measured based on the modified biplane Simpson method. The left atrium volume index [16] was calculated using the biplane area-length method [17]. Dimensions were recorded by standard techniques according to current guidelines [17]. Left ventricular mass index (LVMi) was calculated by the Devereux formula indexed to the body surface area [17]. Conventional transmitral flow was measured with pw-doppler. Early (E), late atrial (A) transmitral peak flow velocities and the ratio (E/A) were measured and three consecutive beats were averaged. Pulsed wave tissue Doppler imaging (TDI) was performed at the junction of the septal and lateral mitral annulus and three consecutive beats were averaged. Early diastolic velocities (E'medial, E' lateral) were recorded; the mean value (E' average) from E' at the medial and lateral mitral annulus was determined. Ratios of E/E'medial, E/E'lateral and E/E'(average) were calculated. Diastolic dysfunction was classified according to the common consensus paper of the American and European Society of Echocardiography (ASE, ESC) [18], including comprehensive evaluation of diastolic function with conventional Doppler tissue Doppler techniques. All examinations were performed by two physicians experienced in the technique, and analyses of LVDD were blinded for IR and glucose metabolism status.

### Laboratory analysis

Insulin resistance was assessed by using Homeostasis Model Assessment of Insulin Resistance (HOMA-IR) and Quantitative Insulin Sensitivity Check Index (QUICKI) in subjects without a history of diabetes before inclusion into the study. The HOMA-IR was calculated from the formula: HOMA-IR= fasting glucose (mg/dl) × Insulin (μU/ml)/405 [19]. QUICKI was assessed with the formula: QUICKI = 1/[log glucose (mg/dL)+log insulin (μU/mL)] [20]. The lower limit of the top quartile of HOMA-IR distribution (i.e. 3.217) was chosen as the threshold for IR.

### Statistical analysis

All analyses were performed using SPSS statistical software (SPSS 17.0, Chicago, IL). The data are presented as median (interquartile range) for continuous variables or absolute number (%) for categorical variables unless otherwise specified. A P value < 0.05 was considered statistically significant and adjusted for multiple comparisons using Bonferroni adjustment. Non-parametric tests for group differences between categories of IR and glucose metabolism disorders were performed. The Wilcoxon-Mann-Whitney-Test was used for the comparison of two independent groups, and the Kruskal Wallis Test was used for more than two independent samples. The Jonckheere-Terpstra test was used to detect effects across ordered categories. Fisher's Test was used for the comparison of two sets of binary variables, and the χ^2 ^test for the comparison of more than 2 sets of categorical variables. To investigate a possible relation between variables, the Pearson's linear correlation coefficients were calculated. Multivariate analysis of covariance and logistic regression models including variable most predictive for the dependent variables were built.

## Results

### Patients characteristics

We included 208 patients in the study (48% woman, 64 ± 11 years), 57 (27%) of whom had T2DM before inclusion (mean duration of diabetes 9.6 ± 9.7 years). An oGTT was performed in 151 individuals, of whom 64 (31%) had a normal glucose tolerance (NGT), 54 (26%) had impaired glucose tolerance (IGT) and 33 (16%) had a new detected diabetes (ND-T2DM). Overall, 90 (43%) individuals had T2DM at inclusion. A metabolic syndrome (MetS) was diagnosed in 117 (59%) patients according to the amended National Cholesterol Education Program's Adult Treatment Panel III (ATP-III) guidelines[21]. In the MetS group, 46 (39%) had T2DM before inclusion, 33 (28%) had ND-T2DM, 22 (18%) had IGT and 16 (14%) had NGT, whereas in the group without MetS, 57% had NGT, 37% IGT and 6% T2D, respectively (p < 0.001). In the IR group, 25 (66%) patients were classified as having MetS, whereas 34 (32%) without IR had a MetS (p < 0.001). In subjects with IR, the prevalence of obesity, defined as BMI > 30kg/m^2^, was 48% compared to 27% in subjects without IR (p = 0.021). Demographics and clinical variables for patients with or without IR, NGT, IGT, and T2DM are shown in table 1 and medications are shown in table 2.

**Table 1:** Demographics, clinical variables and laboratory parameters in subjects with or without IR and in different degrees of glucose disturbance disorders

Variable	IR (+)	IR(-)	**p-value**^**a**^	NGT	IGT	**T2DM**^**#**^	**p-value**^**a**^
**n (%)**	38 (18)	113 (54)	-	64 (31)	54 (26)	90 (43)	-
**Variables**							
Age (years)	62 (53-72)	65 (56-72)	0.604	62 (54-69)	61 (51-70)	71 (63-75)	< 0.001*
Female sex, n (%)	17 (45)	53 (47)	0.484	30 (46)	23 (42)	46 (51)	0.606
WC (cm)	103 (97-118)	100 (92-111)	0.031*	99 (90-110)	100 (95-111)	109 (98-117)	< 0.001*
HC (cm)	105 (98-114)	99 (92-109)	0.019*	100 (91-109)	99 (95-110)	109 (100-116)	< 0.001*
BMI (kg/m^2^)	28 (26-35)	26 (24-30)	0.001*	25 (24-30)	26 (24-31)	29 (26-32)	< 0.001*
SBP, mmHg	135 (128-142)	130 (120-137)	0.020*	128 (120-135)	130 (126-140)	136 (130-142)	< 0.001*
DBP, mmHg	80 (75-82)	80 (70-82)	0.863	80 (70-80)	80 (74-84)	80 (73-84)	0.055
MAP (mmHg)	96 (91-101)	96 (90-100)	0.258	94 (87-98)	96 (91-101)	98 (93-102)	0.003*
Puls-Pressure	57 (48-60)	50 (42-58)	0.031*	50 (41-58)	52 (44-60)	54 (50-61)	0.005*
**CV risk factors**							
HTN, n (%)	33 (89)	95 (84)	0.319	49 (76)	47 (89)	84 (95)	0.002*
HLP, n (%)	23 (61)	73 (64)	0.396	37 (58)	36 (67)	62 (70)	0.305
Smoker, n (%)	2 (5)	21 (19)	0.037*	12 (19)	10 (19)	10 (12)	0.366
Family Hx CAD	24 (63)	59 (52)	0.162	29 (45)	35 (64)	42 (27)	0.065
Hx Stroke, n (%)	0 (0)	4 (3)	0.309	3 (5)	1 (2)	4 (4)	0.674
**Labaratory data**							
Fasting glucose	100 (105-118)	92 (86-98)	< 0.001*	89 (84-94)	104 (90-112)	105 (98-118)	< 0.001*
2h-PG (mg/dl)	183 (138-233)	132 (112-161)	< 0.001*	117 (99-130)	156 (139-164)	225 (212-248) ^b^	< 0.001*
Insulin (μU/L)	18.5 (15.7-24.0)	5.0 (3.0-7.0)	< 0.001*	5.0 (2.0-7.0)	8.0 (5.0-15.0)	9.5 (6.0-18.0) ^b^	< 0.001*
HOMA-IR	5.09 (3.96-6.61)	1.2 (0.69-1.77)	< 0.001*	1.10 (0.44-1.56)	1.86 (1.10-3.85)	2.53 (1.62-4.78) ^b^	<0.001*
QUICKI	0.30 (0.29-0.31)	0.37 (0.35-0.40)	<0.001*	0.38 (0.35-0.44)	0.35 (0.31-0.38)	0.33 (0.30-0.35) ^b^	0.001*
HbA1c (%)	5.9 (5.7-6.3)	5.7 (5.5-6.1)	0.033*	5.7 (5.4-6.0)	5.8 (5.6-6.1)	6.6 (6.0-7.4)	<0.001*
LDL-Ch (mg/dl)	114 (99-136)	109 (90-130)	0.207	109 (92-136)	115 (99-130)	103 (77-130)	0.095
HDL- Ch (mg/dl)	49 (38-57)	54 (4566)	0.044*	54 (46-68)	53 (47-63)	46 (38-59)	0.001*
Total- Ch (mg/dl)	204 (177-222)	191 (171-222)	0.529	189 (175-228)	200 (173-220)	188 (63-221)	0.300
Triglyceride	142 (100-202)	124 (90-169)	0.234	120 (89-157)	126 (95-165)	162 (118-258)	0.001*
Lipoprotein (a)	13 (5-27)	14 (5-39)	0.729	11 (5-35)	15 (4-38)	5 (13-35)	0.944
Creatinine (mg/dl)	0.90 (0.80-1.20)	0.89 (0.77-0.98)	0.253	0.88 (0.75-0.96)	0.90 (0.76-1.0)	0.79 (0.90-1.05)	0.643
hsCRP (mg/dl)	0.2 (0.1-0.5)	0.2 (0.1-0.6)	0.961	0.2 (0.10-0.8)	0.2 (0.1-0.45)	0.3 (0.2-0.6)	0.113

Values are median (interquartile range) or n (%).^a ^Mann-Whitney-Test, Jonckheere-Terpstra test, Fisher or χ^2 ^Test were used as appropriate, ^b ^= 33 subjects with new detected diabetes * statistically significant (p < 0,05). ^# ^Subjects with diabetes before inclusion or new detected diabetes. BMI = Body mass index, CAD = Coronary Artery Disease, Ch = Cholesterol, CV = Cardiovascuklar, DBP = Diastolic blood pressure, HC = Hip circumference, HLP = Hyperlipidaemia, hsCRP = High sensitive CRP, HTN = Hypertension, Hx = history of, IR = insulin resistance, MAP = mean arterial pressure, PG = Postprandial glucose, SBP = Systolic blood pressure, WC = Waist circumference

**Table 2:** Medications

Variable	IR (+)	IR(-)	**p-value**^**a**^	NGT	IGT	**T2DM**^**5#**^	**p-value**^**a**^
n (%)	38 (18)	113 (54)	-	64 (31)	54 (26)	90 (43)	-
**Glucose lowering treatment**							
Metformin, n (%)	0 (0)	1 (0.9)	0.748	0 (0)	1 (2)	64 (71)	<0.001*
Sulfonylurea, n (%)	0 (0)	1 (0.9)	0.748	0 (0)	0 (0)	1 (1)	<0.001*
Insulin therapy, n (%)	0 (0)	0 (0)	-	0 (0)	0 (0)	24 (34)	<0.001*
Thiazolidinediones, n (%)	0 (0)	0 (0)	-	0 (0)	0 (0)	2 (2)	0.266
Glinide, n (%)	0 (0)	0 (0)	-	0 (0)	0 (0)	2 (2)	0.266
**Hypertension treatment**							
Beta-blocker, n (%)	23 (61)	72 (63)	0.434	33 (52)	39 (72)	62 (69)	0.033*
AT1 receptor blocker, n (%)	5 (13)	16 (14)	1.000	9 (14)	6 (11)	15 (17)	0.653
ACE-inhibitor, n (%)	24 (36)	65 (58)	0.573	31 (48)	37 (69)	55 (61)	0. 076
Calcium antagonist, n (%)	7 (18)	18 (16)	0.801	9 (14)	6 (11)	23 (26)	0.055
Diuretics, n (%)	12 (32)	31 (27)	0.679	16 (25)	16 (30)	31 (34)	0.450
**Other Medications**							
Nitrate, n (%)	5 (13)	7 (6)	0.181	1 (2)	7 (13)	16 (18)	0.008*
Acetyl salicylic acid, n (%)	24 (63)	76 (68)	0.691	38 (60)	40 (74)	65 (73)	0.169
Clopidrogel, n (%)	11 (29)	34 (31)	1.000	13 (21)	18 (33)	28 (32)	0.259
Allopurinol, n (%)	5 (13)	6 (5.4)	0.147	3 (5)	3 (6)	15 (17)	0.022*
Aldosterone antagonist, n (%)	1 (3)	0 (0)	0.248	0 (0)	1 (2)	0 (0)	0.245
Statin, n (%)	16 (42)	60 (53)	0.423	27 (42)	28 (52)	49 (55)	0.598

Values are median (interquartile range) or n (%).a Wilcoxon-Mann-Whitney-Test, Jonckheere-Terpstra test, Fisher or χ2 Test were used as appropriate, * statistically significant (p < 0,05). #Subjects with diabetes before inclusion or new detected diabetes based on oGTT results

### Overall prevalence of diastolic dysfunction

The parameters of cardiac assessment are presented in table 3. One hundred seventy (82%) patients had evidence of LVDD of any degree according to the recent guidelines criteria publishes by Nagueh et al. [18]. A more severe form of LVDD (LVDD grade II or grade III, pseudonormal pattern) was observed in 58 (28%) patients, whereas 112 (54%) subjects had mild LVDD (grade I) and 38 (18%) patients had normal diastolic function. In subjects with LVDD of any grade, the BMI (27 [25-32] kg/m^2^) and the waist-circumference (103 [96-115] cm) was significantly higher compared to those without LVDD (BMI 25 [23-30] kg/m^2 ^and waist circumference 98 [88-108] cm, p = 0.022 and p = 0.009, respectively).

**Table 3:** Parameters of cardiac assessment in IR(+) or IR(-), in subjects with normal glucose metabolism and in different degrees of glucose metabolism disturbances.

Variable	IR (+)	IR(-)	**p-value**^**a**^	NGT	IGT	**T2DM**^**5#**^	**p-value**^**a**^
n	38 (18)	113 (54)	-	64 (31)	54 (26)	90 (43)	-
**Diastolic function**							
Normal DF, n (%)	3 (8)	32 (28)	-	24 (38)	8 (15)	6 (7)	-
LVDD (any degree), n (%)	35 (92)	81 (72)	0.013*	40 (63)	46 (85)	84 (93)	0.001*
LVDD grade I, n (%)	23 (60%)	54 (48)	0.015*	29 (45)	34 (63)	49 (54)	0.001*
LVDD grade II, n (%)	12 (32)	27 (24)	0.017*	11 (17)	12 (22)	35 (39)	0.001*
IVS (mm)	11(11-13)	11 (10-13)	0.378	11 (10-13)	11 (10-13)	12 (11-14)	0.012*
**Echocardiography**							
PLW (mm)	12 (9-12)	11 (10-13)	0.617	11 (9-13)	11 (10-13)	12 (10-14)	0.053
LVEDD (mm)	47 (43-49)	44 (40-48)	0.084	45 (41-48)	44 (40-49)	44 (39-48)	0.264
LVESD (mm)	30 (27-35)	30 (26-34)	0.984	31 (28-36)	29 (26-34)	28 (24-33)	0.037*
RWT	0.48 (0.44-0.58)	0.51 (0.43-0.62)	0.625	0.49 (0.40-0.58)	0.48 (0.44-0.59)	0.53 (0.46-0.64)	0.012*
LVM (g/m^2^)	87 (68-109)	84 (69-104)	0.900	83 (69-100)	84 (68-107)	90 (69-111)	0.430
LA- Index (ml/m^2^)	31 (25-38)	30 (24-36)	0.555	28 (24-32)	31 (25-37)	32 (27-38)	0.001*
EF biplan (%)	70 (63-73)	67 (62-71)	0.119	65 (61-70)	67 (63-73)	67 (62-73)	0.205
Smax (cm/s)	6.40 (5.70-7.10)	6.25 (5.50-7.15)	0.883	6.3 (5.6-7.3)	6.3 (5.4-7.0)	6.0 (5.3-7.1)	0.246
VE (cm/s)	70 (60-80)	60 (50-70)	0.155	60 (50-70)	60 (50-80)	70 (60-90)	0.012*
VA (cm/s)	80 (60-90)	70 (60-85)	0.115	70 (55-85)	70 (60-90)	80 (70-90)	<0.001*
VE/VA	0.8 (0.7-1.1)	0.9 (0.7-1.1)	0.566	0.9 (0.8-1.2)	0.9 (0.7-1.1)	0.8 (0.7-1.1)	0.218
E ' septal (cm/s)	5.7 (4.7-7.1)	6.1 (5.3-7.9)	0.099	6.5 (5.3-8.2)	6.2 (5.3-7.6)	5.6 (4.9-6.6)	0.001*
A ' (cm/s)	9.2 (8.0-10.2)	9.6 (8.4-10.4)	0.644	9.7 (8.7-10.4)	9.5 (8.6-10.4)	9.0 (7.810.2)	0.028*
E ' lateral (cm/s)	8.3 (6.5-9.1)	8.8 (7.0-10.6)	0.085	9.4 (7.3-10.9)	8.4 (6.6-10.2)	7.7 (6.5-9.2)	0.002*
Overage E'	7.3 (5.4-7.9)	7.5 (6.2-9.1)	0.061	7.9 (6.3-9.5)	7.3 (5.7-8.7)	6.8 (5.6-7.8)	<0.001*
E'/A' septal	0.6 (0.5-0.8)	0.7 (0.6-0.8)	0.102	0.7 (0.6-0.8)	0.7 (0.6-0.8)	0.7 (0.5-0.7)	0.087
E/E' septal	11.3 (9.6-15.1)	9.8 (7.7-12.7)	0.014*	8.9 (7.3-11.9)	10.2 (8.5-14.1)	12.2 (9.6-15.3)	<0.001*
Overage VE/E'	9.8 (8.3-11.5)	8.1 (6.6-11.0)	0.011*	7.6 (6.2-10.1)	8.8 (7.4-11.0)	10.5 (8.1-13.2)	<0.001*
PVsys (cm/s)	59 (50-66)	60 (51-67)	0.380	59 (50-65)	60 (51-68)	60 (55-68)	0.089
PVdia (cm/s)	46 (38-53)	43 (38-55)	0.839	44 (39-54)	43 (36-52)	46 (40-55)	0.468
PVsys/PVdia	1.2 (1.1-1.5)	1.3 (1.1-1.6)	0.346	1.3 (1.1-1.5)	1.4 (1.1-1.6)	1.4 (1.1-1.6)	0.325
PVa-max (cm/s)	31 (25-34)	31 (28-35)	0.240	31 (28-35)	31 (26-34)	32 (28-35)	0.382
GLS_Avg (-%)	19.3 (21.8-17.0)	19.5 (22.0-17.3)	0.812	20 (22-18)	19 (22-17)	19 (21-17)	0.363
**Cardiac assessment**							
CAD, n (%)	23 (60)	62 (55)	0.339	26 (41)	37 (69)	57 (64)	0.003*
Hx. CABG	1 (3)	5 (5)	0.437	2 (3)	2 (4)	8 (9)	0.223
Hx. PTCA	14 (37)	46 (41)	0.412	14 (22)	28 (52)	43 (49)	0.001*
Hx. MI, n (%)	10 (26)	26 (23)	0.416	7 (11)	19 (35)	16 (18)	0.004*
NYHA (mean, SD)	2.0 (± 0.61)	1.9 (± 0.61)	0.337	1.8	1.8	2.0	0.249
NT-pro-BNP (pg/ml)	(143 ± 105-228)	131 (74-280)	0.916	116 (65-123)	126 (75-114)	152 (80-417)	0.283

CAD = coronary artery disease, CABG = coronary artery bypass graft, DF = diastolic function, GLS = global longitudinal strain, Hx = history off, IVS = intraventricular septum, LA= left atrium, LVEDD = left ventricular enddiastolic diameter, LVESD = left ventricular endsystolic parameter, EF = ejection fraction, LVMi = left ventricular mass index, E '= early diastolic tissue doppler velocity, PVsys = systolic pulmonary vein flow velocity, PVdia = diastolic pulmonary vein flow velocity, PTCA = Percutaneous transluminal coronary angioplasty, PLW = posterolateral wall, RWT = relative wall thickness, SD = Standard deviation, Smax = maximal systolic velocity at the septal mitral annulus, VA = late diastolic transmitral inflow velocity, VE = early diastolic transmitral inflow velocity

### Diastolic function and glucose metabolism

The prevalence of LVDD increased with impaired glucose metabolism (table 3). The highest prevalence was found in those with T2DM as compared to those with NGT (93% vs. 62%, p < 0.001) and those with IGT (93% vs. 85%, p = 0.147). The prevalence was similar in subjects with long standing T2DM and new detected T2DM based on oGTT results (95% vs. 91%, p = 0.665). The status of glucose metabolism remains a significant predictor of LVDD in a logistic regression model adjusted for CAD, hypertension, age, sex, history of previous myocardial infarction, history of previous coronary angioplasty, EF and the oGTT results (p < 0.001).

**Figure 1:**
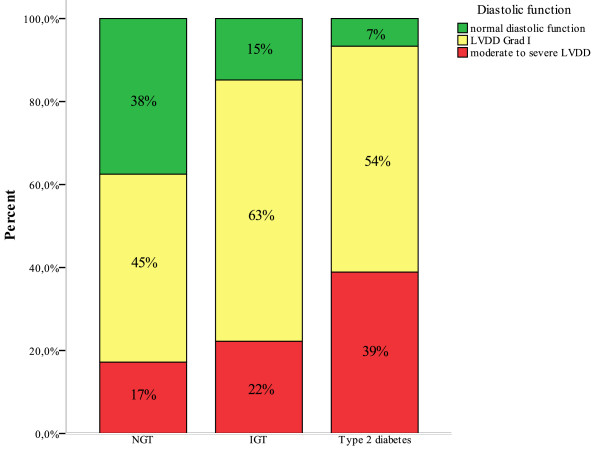
**Diastolic dysfunction and glucose metabolism**. Figure 1 illustrates the prevalence of mild and moderate to severe diastolic dysfunction in patients with normal glucose tolerance (NGT), impaired glucose tolerance (IGT) and type 2 diabetes (T2DM). LVDD = left ventricular diastolic dysfunction

**Figure 2:**
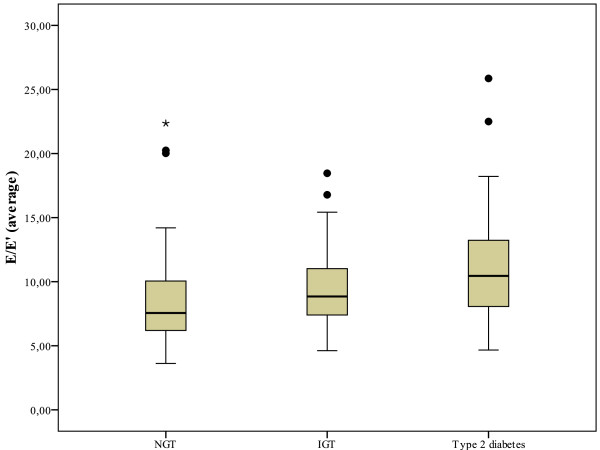
**E/E'average ratio in subjects with normal glucose tolerance, impaired glucose tolerance and diabetes**. Figure 2 illustrates the E/E' ratio in subjects with normal glucose tolerance (NGT), impaired glucose tolerance (IGT) and type 2 diabetes (T2DM).

The prevalence of moderate to severe LVDD (grade II or III) increased with the degree of the glucose metabolism disturbance (p < 0.001, figure 1). In addition, the E/E'(average) ratio, which is indicative for of diastolic dysfunction and elevated left ventricular filling pressures, increases from NGT (7.6 [6.2-10.1]) to IGT (8.8 [7.4-11.0]) and T2DM (10.5 [8.1-13.2]), respectively (p < 0.001, figure 2). Importantly, the E/E'(average) ratio remained significantly higher in the IGT group compared to the NGT group when excluding patients with overt diabetes (p = 0.017). Furthermore, across the whole cohort, the E/E'(average) ratio correlated significant with the HbA1c (r = 0.150, p = 0.037) and with the two hour postprandial glucose level (r = 0.22, p = 0.008). The E/E'(average) ratio in patients above the lower limit of the top quartile of HbA1c distribution (>6.55%) was 10.2 [8.2-13.2] vs. 7.7 [6.2-10.5] in subjects below the upper limit of the lowest quartile (HbA1c < 5.60%, p = 0.001). Similar, the E/E'septal ratio was significantly higher in patients in the upper quartile group compared to subjects in the lowest quartile group (12.1 [10.0-14.5] vs. 9.1 [7.1-12.0], p = 0.001).

Among other echocardiographic parameter used as criteria for the presence of LVDD, the glucose metabolism was associated with the LAi, E'septal, E'lateral and E'average. The LAi increases from NGT to IGT and T2DM (p = 0.001), whereas E'septal, E'lateral and E'average decreases (p = 0.001, p = 0.002, p < 0.001, table 3), respectiverly.

### Diastolic function and HOMA-IR

**Figure 3:**
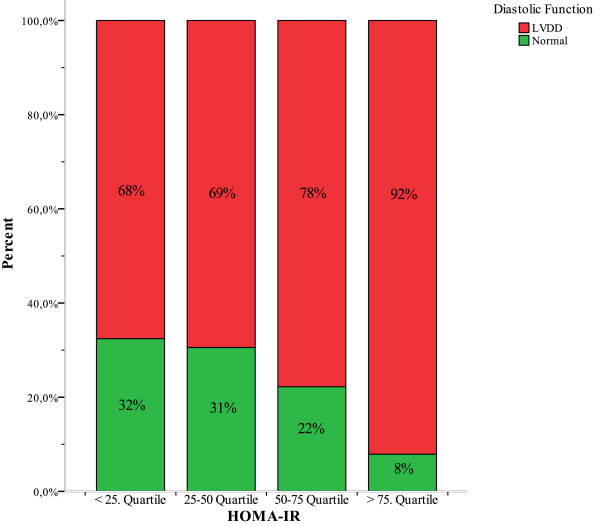
**HOMA-IR Quartile and the overall prevalence of diastolic dysfunction**. Figure 3 illustrates the prevalence of LVDD in relation to different quartiles of the HOMA-IR. LVDD = left ventricular diastolic dysfunction

38 subjects had IR, defined as an HOMR-IR above the lower limit of the top quartile of HOMA-IR distribution (>3.217), 92% of whom had evidence of LVDD, whereas 72% of subjects with a HOMA-IR below this threshold (n = 113) had evidence of LVDD (p < 0.001). The prevalence of LVDD increases along the quartile range of the HOMA-IR (figure 3, p = 0.048). The prevalence of mild or moderate to severe LVDD (grade II or grade III) was 61% and 32% in the IR group vs. 48% and 24% in the non IR group, respectively (χ^2 ^in a 2 × 3 table, p = 0.036).

In subjects with LVDD of any grade, the HOMA-IR was 1.71, [1.04-3.88] vs. 1.09 [0.43-2.2] in subjects with normal diastolic function (p = 0.003), and the QUICKI in the LVDD group was 0.35 [0.31-0.38] vs. 0.37 [0.34-0.44] in individuals without IR (p = 0.005), respectively. The HOMA-IR was independent associated with LVDD on multivariate logistic regression analysis adjusted for CAD, hypertension, age, sex, history of previous myocardial infarction, history of previous coronary angioplasty, EF and history of T2DM before inclusion, a 1-unit increase in HOMA-IR value was associated with an odds ratio for prevalent LVDD of 2.1 (95% CI 1.3-3.1, p = 0.001).

**Figure 4:**
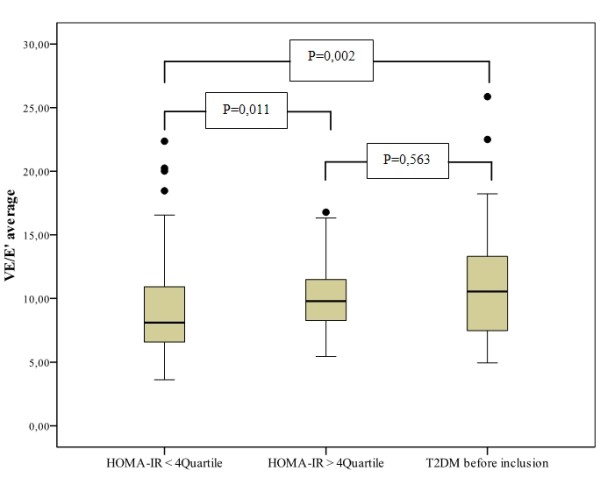
**Average E/E'ratio in subjects with or without insulin resistance or diabetes**. Figure 3 illustrates the E/E'average ratio in subjects with or without insulin resistance (IR) or diabetes.

The E/E'(average) ratio (p = 0.011) and the E/E'septal ratio (p = 0.014) were significantly higher in subjects with IR compared to subjects without IR, both functional parameter indicative for left ventricular diastolic dysfunction with concomitant elevated left ventricular filling pressures (Figure 4). Excluding subjects with a history of diabetes before inclusion, a significant correlation remains between the HbA1c and the E/E'(average) (r = 0.204, p = 0.015) and the E/E'septal ratio (r = 0.188, p = 0.015). In addition, the two hour postprandial glucose level was significantly correlated with the E/E'(average) (r = 0.219, p = 0.008) and the E/E'septal ratio (r = 0.214, p = 0.009). Furthermore, there was a significant correlation between the HbA1c und the LAi (r = 0.185, p = 0.028).

## Discussion

The main finding of the present study is that IR is associated with LVDD independent of overt diabetes. These finding persist after adjustment for CAD, hypertension, age, sex, history of previous myocardial infarction, history of previous coronary angioplasty, EF and glycaemic control. To our knowledge, this is the first study that demonstrated an association in a population of patients without a history of diabetes focusing on the published current guidelines for the diagnosis of LVDD.

Furthermore, our date confirm the observations that T2DM is associated with LVDD [9], which is considered a precursor of diabetic cardiomyopathy. In addition, we were able to extend the findings in previous studies showing the association between LVDD and IGT.

These findings are in line with a limited number of studies that assessed the relationship between prediabetes and LVDD mainly in population based studies [11]. Nevertheless, there are some concerns about methodological issues involving the identification of LVDD patients in the previous studies. The criteria used to define LVDD were highly variable and predominantly did not consider the diagnostic guidelines [22].

Furthermore, most of these studies did not screen for the presence of CAD using coronary angiography. Since CAD has been shown to be associated with LVDD, the lack of information on coronary morphology is a potential source of bias towards an increased prevalence and severity of CAD in patients with LVDD. In contrast, the present study focuses on patient recruitment according to the published current guidelines for the diagnostic workup of LVDD in subjects well characterized for the degree of abnormality in plasma glucose levels and coronary morphology.

We found that IR, as estimated by the HOMA-IR [19], was strongly associated with LVDD in patient without a history of overt diabetes. In a logistic regression model with LVDD as the dependent variable, this association was found independently of established classic risk factors for LVDD such as female sex, age, CAD, hypertension and diabetic state. In addition, high sensitive c-reactive protein (hsCRP) levels did not act significantly to the model. Given the fact that previous studies suggested suggest a link between low-grade inflammation and the presence of LVDD [23,24], it is not likely that low grade inflammation was a significant source of bias in our cohort.

As expected, the presence and degree of a glucose metabolism disorder was another strong predictor for the presents of LVDD. These findings suggest that IR linked to glucose metabolism disorders can contribute to the development of LVDD and that both are important determinants of LVDD. It is well established that diabetes is associated with chronic HF [25,26]. Previous studies have shown that IR, independent of hyperglycemia, predisposes the development of systolic HF [14,27]. In a study by Ingelsson et al. [27], IR was the strongest glucometabolic predictor of chronic HF, even in a subsample without diabetes and independently of other established risk factors for HF. Our study showed, for the first time, that IR is a predictor for the prevalence and severity of LVDD in subjects without overt diabetes. Since, in our study, IR was associated with LVDD, MetS and obesity and LVDD was associated with increased BMI and waist circumference, previous described association between obesity and diastolic HF may be mediated, at least in part, by IR.

This is of clinical relevance, since it has been demonstrated that subtle and subclinical signs of LVDD are associated with an increased cardiovascular morbidity and mortality [3,9,28]. In a study by Wang et al. [29], including subjects with cardiac diseases and controls, a reduced E' velocity was an independent predictor of cardiac death, and From et al. [30] demonstrated an association of increasing E/E' ratio with all-cause mortality in patients with diabetes. Mogelvang et al. [4] showed that, in the general population, LVDD diagnosed by TDI was a powerful and independent predictor of death. Recently, the predictive value of asymptomatic early diastolic dysfunction for HF has been appreciated [29], and asymptomatic early diastolic dysfunction is the most prominent characteristic of diabetic cardiomyopathy [8,31]. Our findings may indicate that the risk for LVDD is already increased in the subclinical phase of glucose metabolism disturbances in subjects with IR, which may precede the development of diabetes. This information would be of clinical importance, because it might strongly justify and encourage the use of therapeutic interventions, including drugs capable of improving insulin sensitivity, with the aim of reducing the risk for diabetic cardiomyopathy.

### Pathophysiological considerations

Although establishing a pathophysiological model linking IR to LVDD is beyond the scope of the present study, several mechanisms for a conditional relationship between IR, glucose metabolism and LVDD should be considered. These mechanism most prominent includes altered insulin signaling, deposition of advanced nonenzymatic glycation end products (AGE) into the ECM [32], increased myocardial collagen deposition with down- regulation of matrix metalloproteinases (MMPs) and upregulation of tissue inhibitors of metalloproteinases (TIMPs) [33], and substrate shifts from glucose to free fatty acids [34] as well as endothelial dysfunction [35].

In our study, the majority of subjects with IR and IGT had a mild form of LVDD (grade I, relaxation abnormalities). Since relaxation is an active, dynamic and energy-consuming myocardial process, impaired relaxation may be due to a reduction in the energy supply. The above mentioned abnormalities in the free acid metabolism may be important contributors to the abnormal myocardial relaxation in subjects with IR. High levels of free acids lead to an inhibition of glucose oxidation, resulting in reduced myocardial ATP availability [36].

In addition, IR can lead to sympathetic nervous system activation [37], which is related to an increased response to angiotensin II [38] and increases the stimulating effects of angiotensin II on collagen production [39], leading to fibrosis and likely subsequent the development of LVDD. Alterations in myocardial structure are usually minimal in the early stages of diabetes and may be partially reversible. As the disease progresses, accumulation of collagen becomes obvious and may play a major role in the development of LVDD [40]. Furthermore, insulin resistance independently influences arterial stiffness [41], and MacIsaac et al [42] demonstrated a link between arterial resistance and diastolic dysfunction in type 2 diabetes, indicating that vascular and LVDD in glucose metabolism disturbances are manifestations of common pathophysiological mechanisms.

Interestingly, even in subjects without a history of diabetes before inclusion into the study, the HbA1c was significantly correlated with the E/E'ratio, a parameter indicative for LVDD with elevated filling pressures. In addition, HbA1c correlated with the LAi, a parameter that indicates long standing LVDD. As the HbA1c incorporates metabolic disturbances over a longer period of time, the LAi reflects a cumulative effect of different contributors to LVDD of longer duration and is less vulnerable to acute changes in preload and afterload, which might have an acute impact on diastolic function. Therefore, the LAi could be labeled as the "HbA1c" of diastolic dysfunction abnormalities.

### Clinical context

Two recent studies, the "ADVANCE" trial [43] and the "ACCORD"- trial [44], reported no significant benefit from intensive HbA1c lowering in terms of cardiovascular outcomes in subjects with long standing diabetes. Similarly, outcomes of recent trials in subjects with HFnEF were frequently disappointing [45-48]. The average duration of diabetes at the start of the "ACCORD" and "ADVANCED" studies ranged from 8 to 11,5 years. Subgroup analysis in "ACCORD" showed that intensive glycaemic control led to fewer cardiovascular complications in diabetic subjects with shorter disease duration and with no antecedent cardiovascular events at baseline. The cardiovascular benefit of intensive glycaemic control in subjects with shorter diabetes duration and no pre-existing cardiovascular disease was also supported by the follow up of the United Kingdom Prospective Diabetes Study (UKPDS) patients [49]. Analogous, the neutral outcome in HFnEF trials might be attributed to the recruitment of patients with advanced diastolic HF and concomitant reduced systolic function, indicating long standing myocardial disease. Likely, therapeutic interventions have failed because the myocardial damage might have become partly irreversible.

Therefore, we speculate that an early intervention is necessary to avoid or reverse LVDD as the first stage in the development of diabetic cardiomyopathy [8,50]. Early treatment strategies should address functional myocardial abnormalities characteristically observed in subjects with diabetes, IR and MetS such as a shift in the myocardial metabolism from glucose to free fatty acids or changes in the ECM turnover. Thiazolidinediones, which are capable to restore glucose utilization, have recently been shown to favorably modify diastolic function as evident from improvement the in E'septal velocity [51]. In this content, one should recognize that physical activity, which can improve insulin sensitivity, was shown to prevent the development of cardiovascular diseases in type 2 diabetes and can improve diastolic function and exercise capacity in subjects with diastolic heart failure [52].

### Limitations

In our study, we did not use the gold standard in the assessment of insulin sensitivity, i.e. glucose clamp [53]. However, previous studies have shown that HOMA-IR is strongly related to clamp-measured insulin resistance in both diabetic and non diabetic subjects [19,54]. Therefore, the HOMA-IR seems to be a reliable diagnostic tool and practicable alternative in the clinical setting in the assessment of IR. Furthermore, the rates of CAD and cardiovascular risk factors were high in this study population. Therefore, the present results may not be readily represent the general population. Nevertheless, association between LVDD, IR and glucose metabolism remains significant after adjustment for CAD and hypertension as covariates into multivariate regression models. Although we based the diagnosis of LVDD on current guidelines which have recently been published [18], their clinical value has yet to be prospectively validated. Lastly, our cross sectional study design does not permit any conclusions on causality.

## Conclusion

The present study suggests that IR and glucose metabolism disorders are independently associated with LVDD, supporting the relevance of LVDD in the development of diabetic cardiomyopathy. Patients with IR and glucose metabolism disorders might represent a target population to prevent the development of HF. Screening programs should address the assessment of diastolic function and therapeutic options capable of improving insulin sensitivity might be considered in the treatment of these patients at risk for the development of heart failure.

## Abbreviations

AGE: advanced nonenzymatic glycation end products; BMI: body mass index; CAD: coronary artery disease; ECM: extracellular matrix; EF: ejection fraction; HF: heart failure; HFnEF: heart failure with normal ejection fraction; HOMR-IR: Homeostasis Model Assessment of Insulin Resistance; IR: insulin resistance; IGT: impaired glucose tolerance; LVDD: left ventricular diastolic dysfunction; LVMI: left ventricular mass index; MetS: metabolic syndrome; MMP: matrix metalloproteinases; ND-T2DM: newly detected type 2 diabetes mellitus; NGT: normal glucose tolerance; oGTT: oral glucose tolerance test; QUICKI: Quantitative Insulin Sensitivity Check Index; TDI: tissue Doppler imaging; TIMP: tissue inhibitors of metalloproteinases; T2DM: type 2 diabetes mellitus

## Competing interests

No potential conflicts of interest relevant to this article were reported

## Authors' contributions

WD wrote manuscript, researched data, performed echocardiographic measurements and statistical analysis. ML reviewed manuscript and contributed to the discussion. WN researched data and contributed to discussion. DS researched data. TS researched data and edited manuscript. FK contributed to the discussion and reviewed manuscript. TK contributed to discussion. RMK contributed to the discussion and reviewed manuscript. MCB contributed to the discussion and reviewed the manuscript. RF researched data and performed echocardiographic measurements. All authors read and approved the final manuscript.
